# Oral Localized Lesion on the Tongue in an Immunocompetent Individual: A Report of a Rare Case With a Comprehensive Review of the Literature

**DOI:** 10.7759/cureus.33469

**Published:** 2023-01-06

**Authors:** Priya Kumar, Sunita Gupta, Aarushi Garg, Aadithya Urs, Jeyaseelan Augustine, Pankaj Sharma, Nita Khurana

**Affiliations:** 1 Oral Pathology and Microbiology, Maulana Azad Institute of Dental Sciences, New Delhi, IND; 2 Oral Medicine and Radiology, Maulana Azad Institute of Dental Sciences, New Delhi, IND; 3 Oral and Maxillofacial Surgery, Maulana Azad Institute of Dental Sciences, New Delhi, IND; 4 Pathology, Maulana Azad Medical College, New Delhi, IND

**Keywords:** oral deep fungal infections, fungal disease, oral histoplasmosis, immunocompetent, histoplasmosis

## Abstract

Histoplasmosis (HP) is a sporadic deep fungal disease that rarely shows oral lesions in various clinical forms. It is usually associated with immunocompromised states, but oral HP has also been reported in many immunocompetent individuals. An unusual case of focal oral HP in a 65-year-old immunocompetent male is reported from New Delhi, India (non-endemic region) presenting with oral ulcerative lesions on the floor of the mouth and lateral surface of the tongue. This case report highlights the importance of prompt diagnosis for the success of the treatment of oral HP along with a thorough review of the literature on HP in immunocompetent patients with oral manifestations. The average age of immunocompetent patients with oral HP is 49.65 years with a marked male predilection. The most common intraoral site is the tongue, followed by the gingiva. Also, five intraosseous cases of HP in immunocompetent patients are reported, among which four are seen in patients from Africa and in a much younger age group (mean: 17.25 years).

## Introduction

Histoplasmosis (HP) or Darling’s disease is a systemic disease caused by a dimorphic fungus, *Histoplasma capsulatum*, which can lead to various clinical manifestations. It exists in the mycelial form at soil temperatures and changes to the yeast form at normal human body temperatures (37°C) [1,2]. It was first described as encapsulated protozoa by Samuel Taylor Darling, a physician in Panama (1906). This disease is highly endemic in the Mississippi and Ohio River basins in the southern United States. Since then, it has been found in temperate climates worldwide [3]. In Indian literature, Panja and Sen (1954) reported the first case of HP in Calcutta [2]. The mode of infection is the inhalation of dust particles from the soil contaminated with bat or bird feces, containing fungal spores, which are the infectious form of the microorganism. Therefore, the lungs are the main portal of entry for fungal spores. HP can present in three forms, the first being the acute pulmonary form in which the spores reach the small bronchioles or alveoli of the lungs. Secondly, *H. capsulatum* may take over the earlier diseased part of the lungs, which becomes structurally defective and produces a chronic HP pulmonary form. The third form usually affects the hosts with compromised immunity known as disseminated HP (DH), a unique and possibly fatal form of the disease [4]. Immunosuppressed patients having a history of human immunodeficiency virus (HIV) infection, organ transplantation, long-duration corticosteroid usage, systemic lupus erythematosus, and Hodgkin's lymphoma have been at risk of dissemination of the disease. Fever, weight loss, weakness, hepatosplenomegaly, and mucocutaneous lesions are the most common manifestations of dissemination [5]. More severe and generalized cases can be observed in patients like the elderly or HIV-positive individuals [1]. Oral lesions are rare and usually seen in DH when present [1]. At times, they may present as the early or the only mucocutaneous appearance of the disease [6].

This article presents a rare case of localized oral HP in an immunocompetent Indian male, along with a thorough review of the literature.

## Case presentation

A 65-year-old male patient reported to the outpatient department with a chief complaint of a non-healing painless ulcer on the tongue for four months. The patient had no history of trauma, pain, pus discharge, or paresthesia. No relevant past dental history was present. The patient had a habit of hand-rolled nicotine cigarette smoking 10-12 times a day for 40 years. The patient was a farmer. The patient had no history of coronavirus disease 2019 (COVID-19) and the vaccination for the same. Medical history was noncontributory.

**Figure 1:**
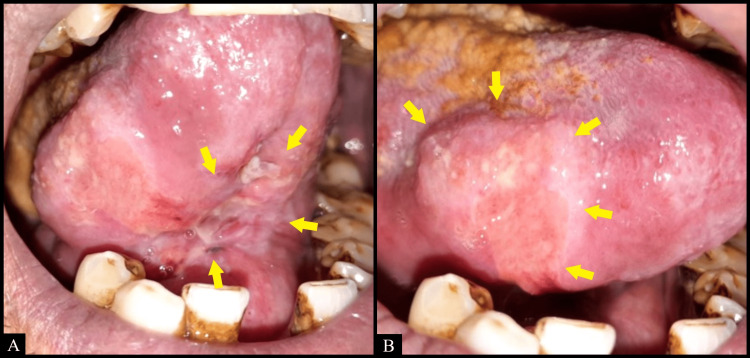
Irregularly shaped ulcer on (A) ventral surface of the tongue as well as the floor of the mouth and (B) right lateral border of the tongue

Intraoral examination revealed an irregularly shaped ulcer on the right lateral border of the tongue extending on the ventral surface of the tongue as well as the floor of the mouth measuring approximately 5 x 4 cm in size (Figure 1). The borders of the ulcer appeared edematous and raised. It was indurated and tender, accompanied by bilateral submandibular lymphadenopathy on palpation. Differential diagnoses of squamous cell carcinoma, traumatic ulcer, and deep fungal infection were considered.

**Figure 2:**
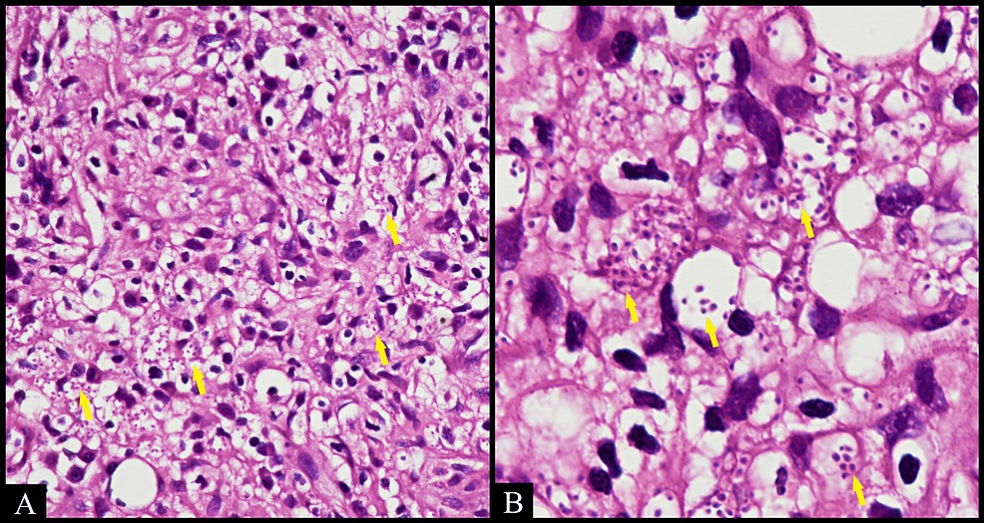
Photomicrograph of the specimen shows (A) numerous yeast cells in vacuolated cytoplasm of macrophage (arrows) (hematoxylin & eosin (HE), 40x). (B) High-power photomicrograph of the specimen showing macrophages with intracellular microorganisms surrounded by a clear halo (arrows) (HE, 100x, oil immersion)

An incisional biopsy was carried out for histopathological analysis and routine hematological examinations. All the hematological parameters were within normal limits. The patient tested seronegative for HIV. The patient's fasting and post-prandial blood sugar levels were within normal limits. The fungal culture from the dorsal surface of the tongue showed candidiasis and the ventral surface showed no growth. The biopsy specimen was not sent for fungal culture. Fungal blood and respiratory culture and imaging were not performed, as the patient was not willing to get them done. Chest X-rays were normal. The histopathological analysis revealed numerous ill-defined granulomatous aggregates consisting of epithelioid cells, lymphocytes, and multinucleated giant cells, some of them showing Langhans-type giant cells with central caseation. Multiple round to oval intracytoplasmic inclusion bodies with a peripheral halo were observed. Multinucleated foreign body giant cells were randomly distributed in loosely collagenous connective tissue and moderate infiltration of lymphocytes and plasma cells. Few granulomas were noted between the skeletal muscle fibers and nerves (Figure 2).

**Figure 3:**
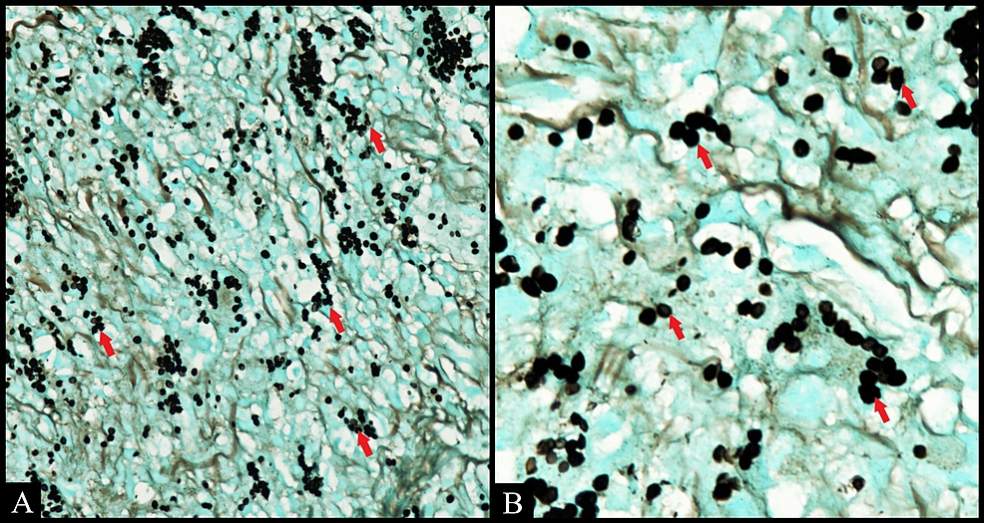
Numerous circular yeast-like cells stained black (arrow) scattered throughout the tissue. (A) Grocott methenamine silver (GMS) (40x). (B) GMS (100x, oil immersion)

**Figure 4:**
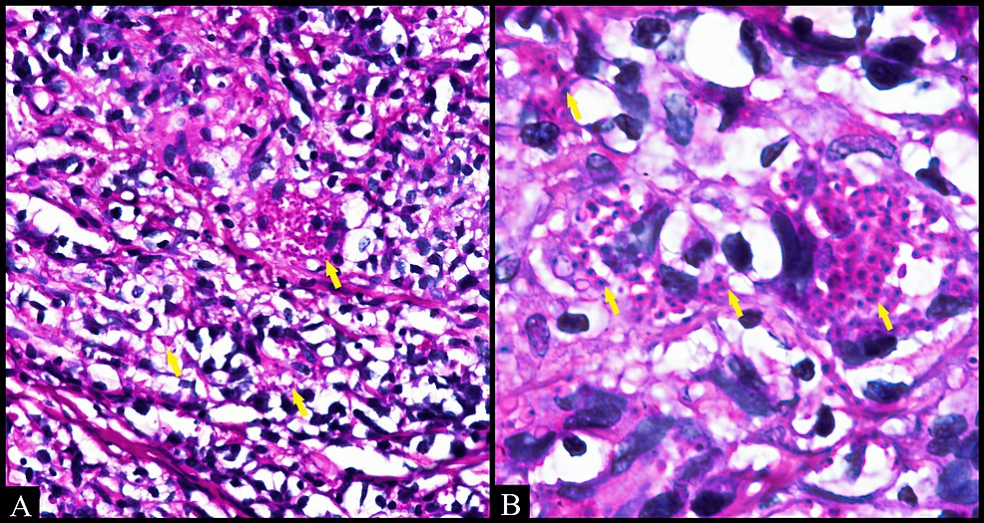
Numerous magenta-colored circular yeast-like cells (arrow) scattered throughout the tissue in clusters and individually. (A) Periodic acid-Schiff (PAS) (40x). (B) PAS (100x, oil immersion)

Grocott methenamine silver (GMS) and periodic acid-Schiff (PAS) staining showed numerous intracytoplasmic fungal organisms throughout the lesional tissue (Figures 3, 4). Acid-fast bacilli (AFB) staining was negative on the biopsy tissue. A final diagnosis of histoplasmosis was arrived at.

**Figure 5:**
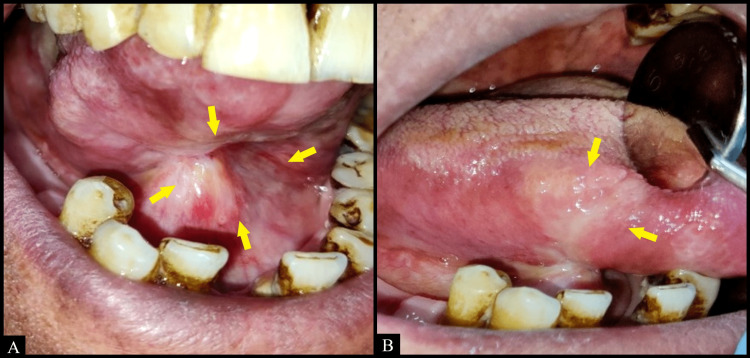
Resolution of ulcer over (A) base of the tongue and (B) right lateral border of the tongue after one month of antifungal therapy

As the patient had only oral involvement, he was treated in the outpatient setting with antifungal drugs. He was given a tablet of itraconazole 200 mg orally thrice daily for three days followed by 200 mg twice daily for 12 weeks. After one month of follow-up, there was a marked resolution of lesions with significant symptomatic improvement (Figure 5). The patient has been under regular follow-up since then.

## Discussion

Review of the literature

A total of 534 English language articles were retrieved from PubMed using the search words "oral histoplasmosis." Out of these 534 articles, we found 37 articles with only 41 cases of localized oral HP in immunocompetent individuals (Table 1) [3-5,7-41]. Our review of the literature suggested that the average age of immunocompetent patients with HP with oral manifestations is 49.65 years (Table 1). There was a marked male predilection with 31 males and 11 females (2.8:1). The most common intraoral site of localized HP in the cases reviewed was the tongue, followed by the gingiva. Interestingly, of the five intraosseous cases of HP in immunocompetent patients (#2, 8, 11, 12, and 21), four were seen in patients from Africa and in a much younger age group (mean: 17.25 years) compared to the average age of 49.65 years. A possible explanation could be the increased prevalence of *Histoplasma duboisii* in tropical Africa [18]. *H. duboisii* differs from *H. capsulatum* as it manifests commonly as lesions of the skin, subcutaneous tissue, and destructive lesions of bone. Due to their young age and aggressive clinical presentation, most of these destructive lesions were initially suspected to be Burkitt’s lymphoma [18].

**Table 1:** Review of the literature M: male; F: female; w: weeks; y: years; mo: months; HE: hematoxylin & eosin; PAS: periodic acid-Schiff stain; GMS: Grocott's methenamine silver stain; Anti-HC Ab: anti-*Histoplasma capsulatum* antibody; HC Ag: *Histoplasma capsulatum* antigen; PCR: polymerase chain reaction; WB: western blotting; CFT: complement fixation test; NM: not mentioned; FCZ: fluconazole; AMB: amphotericin B; ITZ: itraconazole; KCZ: ketoconazole; MCZ: miconazole.

S. No.	Author	Year	Country	Age	Sex	Site	Duration	Diagnostic criteria	Treatment
1	Tiecke R et al. [7]	1963	USA	78	M	Tongue	1 mo	HE, HC Ag	Surgery
2	Akinosi JO [8]	1970	Nigeria	25	F	Mandible	3 w	HE, GMS	IV AMB
3	Banerjee AK et al. [9]	1971	India	48	M	Buccal + labial mucosa + tongue	4 y	HE	IV AMB
4	Talvalkar GV [10]	1972	India	56	M	Tongue	NM	NM	NM
5	Sanyal M et al. [11]	1973	India	59	M	Palate + gingiva + tongue	4 mo	HE, PAS, culture	NM
6	Basler RS et al. [12]	1974	USA	55	F	Tongue	1.5 mo	HE, histoplasmin Ag	IV AMB
7	Reddy CR et al. [13]	1974	India	45	M	Palate + gingiva	NM	NM	NM
8	Daramola JO et al. [14]	1979	Nigeria	13	F	Maxilla	6 mo	HE, PAS, GMS	IV AMB + surgery
9	Nicholls M et al. [15]	1980	Australia	50	M	Gingiva	NM	HE, PAS, culture, HC Ag	Oral MCZ
10	Toth BB and Frame RR [16]	1983	USA	62	M	Palate	8 mo	HE, culture	Oral KCZ
11	Adekeye EO et al. [17]	1988	Nigeria	14	M	Mandible	4 mo	HE	IV AMB + surgery
12	Dobleman TJ et al. [18]	1989	USA	82	M	Mandible	NM	HE, GMS, culture (Neg), HC Ag (Neg)	Oral KCZ
13	Loh F et al. [4]	1989	Singapore	33	F	Gingiva	1.5 mo	HE, PAS, culture	Oral KCZ
14	Fraysse E et al. [19]	1990	France	NM	M	Buccal mucosa	NM	NM	NM
15	Padhye AA et al. [20]	1994	India	30	M	Palate + gingiva	6 mo	HE, GMS, culture, chemiluminescence	NM
16	Mignogna MD et al. [21]	2001	Italy	44	M	Tongue	1.5 mo	HE, PAS, GMS	Oral FCZ
17	Ferreira OG et al. [22]	2002	Brazil	57	F	Tongue	NM	HE, PAS, GMS	IV AMB
18	Ferreira OG et al. [22]	2002	Brazil	26	F	Gingiva	NM	HE, PAS, GMS	IV AMB
19	Rahman MT et al. [23]	2004	Malaysia	40	M	NM	6 mo	HE	NM
20	O'Sullivan MN et al. [3]	2004	Australia	53	F	Gingiva + buccal mucosa	NM	HE, GMS, culture	Oral ITZ
21	N'Golet A et al. [24]	2005	Congo	17	M	Mandible	7 mo	HE, PAS, GMS	IV AMB
22	Alcure ML et al. [25]	2006	Australia	53	F	Gingiva + buccal mucosa	NM	HE, GMS, culture	Oral ITZ
23	Vijayan C [26]	2007	India	47	M	Buccal mucosa	2 mo	HE, GMS, culture (Neg)	Oral ITZ
24	Ge L et al. [27]	2010	China	51	M	Palate	2 mo	HE, PAS, PCR, culture	Oral ITZ
25	Garg A et al. [28]	2010	India	42	M	Tongue	6 mo	HE, GMS	Oral ITZ
26	Wierna A et al. [29]	2010	Brazil	57	M	Tongue	7 mo	HE, HC Ag	Oral ITZ
27	Edwards PC et al. [30]	2010	USA	28	F	Palate + gingiva	NM	HE, PAS, GMS	NM
28	Fortuna G et al. [31]	2011	USA	67	M	Palate	6 mo	HE, anti-HC Ab	Oral ITZ
29	Stander S et al. [32]	2012	Nigeria	35	M	Palate	2 w	HE	NM
30	de Paulo LFB et al. [33]	2013	Brazil	30	M	Tongue	1 mo	HE, GMS	NM
31	de Paulo LFB et al. [33]	2013	Brazil	70	M	Tongue	NM	HE, GMS	NM
32	de Paulo LFB et al. [33]	2013	Brazil	50	M	Tongue	NM	HE, GMS	NM
33	Barket S et al. [34]	2013	USA	47	F	Gingiva	NM	HE, GMS	Oral ITZ
34	Kash N et al. [35]	2014	USA	61	F	Tongue	1 mo	HE, GMS	Oral ITZ
35	Maharaja K et al. [36]	2015	India	40	M	Tongue	4 mo	HE, GMS, culture (Neg)	Oral ITZ
36	Figueira JA et al. [37]	2017	Brazil	62	M	Gingiva	3 mo	HE, GMS	Oral ITZ
37	O'Connell Ferster AP et al. [5]	2018	USA	80	M	Tongue + floor of the mouth		HE, PAS, GMS, anti-HC Ab	Oral KCZ
38	Lindner AK et al. [38]	2018	Germany	55	M	Gingiva + lip	4 mo	HE, PCR, WB, CFT	Oral ITZ
39	Khullar G et al. [39]	2018	India	70	M	Tongue	3 mo	HE., PAS, culture	NM
40	Kumar A et al. [40]	2019	India	67	M	Oral commissure	1 mo	HE, GMS, HC Ag	Oral ITZ
41	Mutalik VS et al. [41]	2020	Canada	72	M	Oral commissure	1 mo	HE, GMS, HC Ag	IV ITZ
42	Kumar P et al. (present case)	2021	India	65	M	Tongue	4 mo	HE, PAS, GMS, culture (Neg)	Oral ITZ

The mean time between the onset of symptoms and clinical presentation of the oral lesions was found to be 5.65 months (two weeks to four years). The most commonly used diagnostic methods in the reviewed literature were histopathology using hematoxylin and eosin staining, followed by GMS, PAS, and culture. Three out of 42 cases showed negative culture results. Most patients were treated with oral antifungal drugs like itraconazole. Intravenous amphotericin B and surgery were other treatment modalities.

Discussion

Histoplasmosis is predominantly a pulmonary disease, and its ecological reservoir is soil [27]. There are two varieties of *H. capsulatum* that are pathogenic to humans, *H. capsulatum var. capsulatum* (endemic in Central and North America) and *H. capsulatum var. duboisii* (predominant in West Africa), and a third variety, *H. capsulatum var. farciminosum*, that is an equine pathogen, which exists in Africa [27,42]. This infection is endemic in certain areas of the USA, Brazil, Indonesia, Australia, and Malaysia, with few cases reported from Gangetic plains, Maharashtra, West Bengal, and Uttar Pradesh in India [40]. The fungus spreads by hematogenous route or direct inoculation into the mucosa.

HP is common in immunocompromised patients; however, many studies have shown a high prevalence of DH in immunocompetent patients too, where it tends to be an asymptomatic or self-limited disease, whereas, in immunocompromised patients, it might disseminate in a possibly fatal course of action. Cell-mediated immunity is the primary defense mechanism in the human body against the organism leading to granulomatous and histiocytic inflammation in the host [42,43].

The reported prevalence of HP in adults 65 years and older is nearly 3.4 cases per 100,000 population [5]. Its severe form commonly affects infants with known immaturity of immune processes and the elderly because of a decline in cell-mediated immunity [25].

According to Padhye et al. [20], HP in Indians occurs mainly in the extrapulmonary sites, especially in the oral cavity. Localized HP may rarely result from direct inoculation of *H. capsulatum* into oral mucosa [40]. Oral lesions along with constitutional symptoms like dysphagia, weight loss, loss of appetite, and irregular low-grade fever suggest a clinical differential diagnosis of squamous cell carcinoma, oral involvement of tuberculosis, syphilitic chancre, granulomatosis with polyangiitis, or major aphthous ulcer [23]. A study by De and Nath has shown DH as a critical cause of pyrexia in immunocompetent patients [42]. Most of those affected are from a rural background (85%) and are mainly male, emphasizing the significance of occupation and exposure to the soil as a hint to the diagnosis of DH [42].

The histological differential diagnosis of HP includes other conditions in which parasitized macrophages are seen, like leishmaniasis. Sections of HP show numerous multinucleated giant cells, epithelioid cell granulomas, and mixed inflammatory cells, including multiple histiocytes stuffed with ovoid yeast forms of *H. capsulatum*, whereas the parasites in leishmaniasis lack the clear halo seen in *H. capsulatum* and tend to aggregate at the periphery of the macrophage (marquee sign). *Leishmania donovani* (LD) bodies can be recognized by a nucleus and bar-shaped kinetoplast within each amastigote and are PAS negative [44].

The definitive diagnosis needs isolation of *H. capsulatum* on special culture media, such as Sabouraud agar, following incubation at 25°C for six to 12 weeks. The fungal colonies appear smooth initially but become cottony, filamentous, and brownish with age. Positive cultures give the most substantial evidence for HP, but most asymptomatic patients have negative cultures. Apart from cultures, skin scrapings, body fluids, and exudates should be analyzed using 10% potassium hydroxide (KOH) and Parker ink or calcofluor white mounts. Tissue sections should be stained using PAS, GMS, or gram stain [45].

Other techniques are also available to supplement a culture and microscopic assessment. Using serology as a marker, antibodies to *H. capsulatum* can be detected by three available methods: complement fixation (CF), immunodiffusion (ID), and enzyme immunoassay (EIA). An antibody titer of 1:32 or greater on the CF test denotes an acute infection. ID test qualitatively measures precipitating (H and M precipitin bands) antibodies. The H band always specifies active infection, while the M band is less specific. The most sensitive test is EIA but shows a high false-positivity rate. In combination with antigen testing, serology improves the diagnostic yield for acute pulmonary HP though serological testing (antigen and antibody) shows a high rate of cross-reactivity with other fungal species. Serial testing of *Histoplasma* antigen and antibodies in urine and serum are good ways to assess response to therapy [46]. Polymerase chain reaction diagnosis based on the amplification of fungal gene sequences is an effective tool for detecting invasive mycoses [45].

The treatment depends on the severity of the infection and the patient’s medical condition, with antifungal drugs being the treatment of choice [47]. In immunocompromised patients such as those with HIV presenting with limited or disseminated disease, intravenous liposomal amphotericin B is the treatment of choice as the regular (amphotericin B deoxycholate) form is less used due to adverse reactions [48]. However, in immunocompetent patients and those with localized disease, itraconazole is the preferred treatment modality as it is safe and has low toxicity compared to amphotericin [49,50]. The prognosis in immunocompetent patients having mild to moderate infections is usually good. However, the prognosis is poor for patients with severe conditions, those who are immunosuppressed or have chronic cavitary pneumonia, meningitis, or fibrosing mediastinitis.

## Conclusions

In conclusion, although localized oral HP in an immunocompetent individual is rare, one must always rule out a hidden immunosuppressive state for optimal treatment outcomes. Also, an HP must be considered in the differential diagnosis of a non-healing oral ulcer despite the absence of pulmonary symptoms. Early diagnosis and management of such a deep fungal disease are crucial to prevent it from getting disseminated throughout the body. On review of the literature, oral HP is most commonly found in elderly males, with the tongue being the most common intraoral site. Such lesions can be frequently mistaken for malignant ulcers, which should always be ruled out before performing any treatment.
